# The effects of exposure to images of others' suffering and vulnerability on altruistic, trust-based, and reciprocated economic decision-making

**DOI:** 10.1371/journal.pone.0194569

**Published:** 2018-03-21

**Authors:** Philip A. Powell, Olivia Wills, Gemma Reynolds, Kaisa Puustinen-Hopper, Jennifer Roberts

**Affiliations:** 1 Department of Economics, University of Sheffield, Sheffield, United Kingdom; 2 School of Health and Related Research, University of Sheffield, Sheffield, United Kingdom; 3 Department of Psychology, Middlesex University, London, United Kingdom; 4 Centre for Advanced Spatial Analysis, University College London, London, United Kingdom; 5 Impossible Labs, London, United Kingdom; Oregon Health and Science University, UNITED STATES

## Abstract

In this paper we explored the effects of exposure to images of the suffering and vulnerability of others on altruistic, trust-based, and reciprocated incentivized economic decisions, accounting for differences in participants’ dispositional empathy and reported in-group trust for their recipient(s). This was done using a pictorial priming task, framed as a memory test, and a triadic economic game design. Using the largest experimental sample to date to explore this issue, our integrated analysis of two online experiments (total *N* = 519), found statistically consistent evidence that exposure to images of suffering and vulnerability (vs. neutral images) increased altruistic in-group giving as measured by the “triple dictator game”, and that the manipulation was significantly more effective in those who reported lower trust for their recipients. The experimental manipulation also significantly increased altruistic giving in the standard “dictator game” and trust-based giving in the “investment game”, but only in those who were lower in in-group trust and also high in affective or cognitive empathy. Complementary qualitative evidence revealed the strongest motivations associated with increased giving in the experimental condition were greater assumed reciprocation and a lower aversion to risk. However, no consistent effects of the experimental manipulation on participants’ reciprocated decisions were observed. These findings suggest that, as well as altruistic decision-making in the “triple dictator game”, collaboratively witnessing the suffering of others may heighten trust-based in-group giving in the “investment game” for some people, but the effects are heterogeneous and sensitive to context.

## Introduction

It is widely accepted that environmental information we are exposed to “in the moment” influences our decisions [1]. In economic decision-making, for example, witnessing the suffering and vulnerability of others can induce affective and empathic reactions that lead to increased altruistic, or other-regarding, behaviour that is antithetical to neoclassical models of financial self-interest [2]. In charitable giving, for example, vivid portrayals of a single suffering individual (vs. anonymised groups) are used to induce a response in the viewer, as a means of encouraging donations [3]. Such exposure effects may operate via a number of channels, including by altering affective states, such as compassion and sadness [4], and increasing the identifiable nature of those in-need (i.e., “the identifiable victim effect” [3]).

Prior work that has looked at the effects of exposure to suffering and vulnerability on economic decision-making has typically shown significant positive effects on other-regarding outcomes. Saslow et al. [5], in his second study for example, used a short video clip about child poverty (vs. a neutral video) in an attempt to alter economic behaviour. The researchers found positive effects of exposure to the child poverty video on hypothetical amounts given in the dictator game and a measure of increased support for charitable donations. Furthermore, these effects were driven by a subgroup of participants who identified as less religious. Another recent study [6], using an adapted dictator game, showed that participants gave more money to people featured in videos depicting suffering (vs. neutral videos). To our knowledge, however, no research has explored the effects of exposure to stimuli depicting the suffering and vulnerability of others on different kinds of economic decisions (e.g., those involving self-invested trust and reciprocation [7]).

Another important insight from the literature is that the effects of exposure to the suffering of others may be heterogeneous [8], with results varying based on underlying traits (such as religiosity [5]). Two potentially important, unexplored factors include people’s dispositional empathy and trust. Individual differences in empathy—operationalised as the capacity to recognise and feel others’ emotional states—have separable cognitive and affective components. Cognitive empathy, or perspective-taking, refers to the ability to identify, recognise, and infer others’ feeling states, while affective empathy describes the capacity to feel or share those states [9]. Both of these constructs may be important for determining people’s distributive economic behaviour.

Mounting evidence suggests a link between empathy and other-regarding economic decisions. Edele, Dziobek, and Keller [10] found that affective empathy, in response to photos displaying affective states, had a stronger effect than cognitive empathy on altruistic donations in a dictator game (but see [11]). Trait “empathic concern”—where the boundaries between empathy and compassion blur—has also been related to charitable giving in large secondary data, with perspective-taking displaying mixed effects [12]. Barraza and Zak [13] found that exposure to an emotional video, featuring a child with terminal cancer, produced more generous monetary offers towards a stranger in the ultimatum game. Finally, encouraging cognitive empathy by asking participants to “put themselves in the other’s shoes” has been shown to lead to increased giving in the dictator game [14]. One of the drawbacks of this work is that it has considered people’s underlying capacity for empathy, or exposure to evocative stimuli, in isolation, rather than modelling the potential interaction between the two.

Alongside other-regarding preferences, trust has long been identified as a key motivator of economic behaviour [15]. Self-reported trust for strangers is significantly related to decisions in the dictator game [16]; behavioural trust decisions in the investment game [17]; and economic trustworthiness [18]. People consistently give more in economic games that measure trust (with a self-invested return) than in altruistic games (with no expected return; e.g., [19]). To our knowledge, no research to date has considered the effect of exposure to others’ suffering on subsequent trust decisions. As well as enhancing altruistic generosity, affective responses to stimuli depicting suffering and vulnerable others, such as compassion, may prime cooperation and affiliation [4], trust and reciprocated decisions. Equally, trust decisions require a (implicit or explicit) prediction of how the other party is likely to behave, which may be altered through collaborative exposure to stimuli depicting vulnerability and suffering.

The present research was designed to extend and improve upon past research by using a triadic game design to: i) test whether the effects of priming themes of suffering and vulnerability are restricted to other-regarding economic outcomes, or extend to trust-based and reciprocated decisions; ii) explore whether results are consistent across different types of altruistic giving task; and iii) explore the moderating effects of dispositional (cognitive and affective) empathy and trust. We conducted two large decision-making experiments online. In the first, participants received exposure to pictures depicting suffering and vulnerability (vs. neutral pictures), framed as a memory test, before taking part in a triad of economic games with a stranger, designed to separate out altruistic, trust-based, and reciprocated decision-making [19]. Participants also completed measures of dispositional empathy and trust. In the second experiment we replicated this paradigm in an independent sample with modified elements of the study design. To estimate more accurate and stable effects, and to test statistically which effects replicated across the two experiments, we combined these data for analysis, following the procedures outlined in integrative data analysis [20–21].

Based on past research (e.g., [13]), we hypothesised that participants who received the pictures depicting suffering and vulnerability (vs. neutral pictures) would give more in economic games designed to measure altruistic giving (i.e., the “dictator” games). However, based on the potential affiliative effects of being exposed to suffering and vulnerable others [4], we hypothesised that we may observe additional effects of the experimental exposure on trust-based and reciprocated decisions, when controlling for other-regarding behaviour (i.e., in the “investment game”). We also expected to observe positive main effects for dispositional empathy and trust on economic decisions (e.g., [14, 16]), with affective empathy having a stronger effect on altruistic decisions [10], and cognitive empathy being more important in strategic (investment) decisions [22]. Finally, we explored whether dispositional empathy and trust moderated the effects of exposure to pictures depicting suffering and vulnerability on economic behaviour.

## Materials and methods

### Experiment 1

#### Participants

Of 387 staff and students at the host institution who completed an initial sign-up survey, 320 volunteers (199 women) completed the online experiment. Three of these volunteers were excluded as outliers, taking longer than *M* + 3 *SD* in time to complete the study, giving a final sample of 317 participants (197 women). Participants’ ages ranged from 18 to 77 years, with a mean of 26.97 (*SD* = 9.45). The majority were students (*n* = 214), and UK nationals (*n* = 253). The experimental subsample (*n* = 317) did not differ significantly on any observed variable from those who did not participate (*n* = 70). Participants were paid a fixed rate (£2.00), plus a variable amount ranging from £0.00 - £7.00 in Amazon.co.uk e-credit, dependent on their decisions and the decisions of another participant (see Procedure).

#### Materials

For the experimental manipulation, static images depicting suffering and vulnerability were presented to participants assigned to the experimental condition. These stimuli were developed by Oveis et al. [23], and have been shown to reliably alter participants’ affective profiles, including other-regarding emotions, such as compassion [24]. Fifteen images depicting vulnerability, helplessness, and physical and emotional pain (e.g., babies crying, homeless, starving children) were displayed to participants for 8 seconds each. In the control condition, participants viewed 15 neutral images (taken from [23]) for the same duration. These images depicted neutral objects, scenes, and shapes not designed to induce any affective response in particular.

For the economic games we used a modified version of the triadic design by Cox [19], adapted for repeated measures by Ashraf et al. [18]. This design separates decisions based on other-regarding preferences (altruism), from those involving self-invested trust and reciprocity. Participants played three economic games in a counterbalanced order. In each game, participants were given 10 experimental “currency units” (CU; 1CU = 10p) and asked how they would like to distribute them. Participants played these games alone, but were told their responses would be randomly and anonymously paired with another participant from the same institution. In Game A (the “dictator game” [DG]), participants were told that their partner had 0 CU, and they could choose to give any or none of their CUs to their partner. Game B (the “triple dictator game” [TDG]) was identical to Game A, but participants were told that any amount they chose to give to the other player would be tripled by the experimenter. Game C (the “investment game” [IG]) was identical to Game B, but participants were told that the other player would subsequently have the opportunity to return a proportion of the tripled endowment back to the participant. In the IG, participants were also asked the amount they *expected* the other player to return, and, using a strategy method, how much they would reciprocate given each potential offer in the IG [18].

#### Measures

Participants’ dispositional empathy was measured using the Questionnaire of Cognitive and Affective Empathy (QCAE) [9]. For each of 31 items, participants rated their agreement on a 4-point Likert scale (1 = *strongly disagree*, 4 = *strongly agree*). Nineteen items assessed cognitive empathy, and twelve items measured affective empathy. The internal consistencies of the cognitive (α = .89) and affective (α = .78) empathy subscales were good.

We used an adapted version of the English-version of the German Socio-Economic Panel (SOEP) Trust scale [25] to measure trust for the participants’ institutional in-group partner in the economic games. The SOEP-Trust has been shown to be an internally consistent and valid measure of trust in strangers [25]. We adapted the SOEP-Trust by changing the object to “staff/students at the host university” (e.g., “In general, you can trust staff/students at the host university”). For each of three items, participants rated their agreement on a 4-point Likert scale (1 = *disagree strongly*, 4 = *agree strongly*). The internal reliability of this adapted trust measure (α = .73) was adequate.

Five items were used to measure participants’ affective state post-manipulation. For each of five emotional adjectives (happy, sad, disgusted, proud, and compassionate), participants responded to the question “How do you feel right now?” on a 4-point Likert scale (1 = *not at all*, 4 = *very*).

Following the experimental manipulation, participants were asked five multiple-choice memory questions about the images they had viewed (e.g., “There were images showing a baby crying”, *true/false*; “One of the images showed a mug on a table. What colour was the mug?” *Blue / pink / red / green*). Participants did not receive any feedback.

We present data on the following economic decisions. *DGgive*, *TDGgive*, and *IGgive* are the amounts participants opted to give in the DG, TDG, and IG, respectively, as ordinal integers from 0 to 10 (i.e., 0 = gave nothing, 10 = gave everything). *IGexpect* is the proportion (.00–1.00) participants said they expected their partner to return in the IG (if the value of IGgive was 0, IGexpect was also coded as 0). Finally, *IGreciprocate* is the average proportion (.00–1.00) participants said they would return across offers in the IG.

In our regression models we controlled for observed variables that we expected, *a priori*, may have a significant relationship with economic decisions, including participants’ gender (0 = male, 1 = female), age, nationality (0 = UK, 1 = international), employment status (0 = student, 1 = staff), whether they had an economics affiliation (0 = no, 1 = yes), time taken to complete the experiment, and the order of economic gameplay.

#### Procedure

Ethical approval was granted from the University of Sheffield’s Research Ethics Committee (project 003334) prior to data collection. Written informed consent was obtained from participants. The invitation to participate was disseminated to staff and students by e-mail. In the initial invitation, volunteers were sought for an “online study on economic decisions”, in which they would earn a base rate and a variable amount depending on their responses, to be paid as an Amazon.co.uk gift certificate. Participants were first provided with a link to an online sign-up survey, hosted on Qualtrics (www.qualtrics.com). In this survey, participants provided their consent, university e-mail address, demographic information, and completed the QCAE [9]. Participants were then stratified on age, gender, and trait empathy, and block-randomised to the experimental or control condition based on randomised lists.

Signed-up volunteers were e-mailed one week later with a (experimental or control) link to the experiment. In order to reduce demand characteristics, and maximise attention to the experimental manipulation, volunteers were informed the study was about “the relationship between memory and economic decision-making”, that they would be shown images and asked some questions about them. Participants were informed that they would then play economic “games”, which had “financial consequences” for them and another person, as their responses would be randomly paired with another individual at the host institution who also participated (full instructions for the experimental games are provided in S1 Text).

Following the experimental manipulation, participants completed the 5-item state mood measure and responded to the memory questions, before playing the games in a counterbalanced order. Participants were told that they were “player 1” in all three economic games (except in the IG, where they were also asked what they would do *if* they were “player 2” for reciprocated decisions). They were told that they would respond to the games on their own, but their responses would be randomly and anonymously paired with another participant to calculate their payment. Lastly, participants answered the questions on trust, and were debriefed. Participants’ study payments were calculated by randomly pairing them with another participant and then, for each of the games, randomly allocating one of each pair as player 1 or player 2, then summing to produce a payment.

### Experiment 2

The second experiment was designed to replicate and improve the design of Experiment 1 in an independent sample. In doing so, several design caveats were addressed. First, in Experiment 1 participants were told that they would be *randomly* matched to another participant *following* the study, and so may have interpreted their payments as being randomly, rather than strategically, determined. Second, the trust measure in Experiment 1 was administered *after* the economic games, and while it did not differ across conditions, may have been affected (homogeneously) by gameplay. Third, no data were collected on participants’ decision-making strategies in Experiment 1, resulting in their motivations being somewhat of a “black box”. Accordingly, a second experiment was designed to assess the robustness of the first in another sample, with a procedure that improved on these caveats.

#### Participants

Of 273 staff and students at two host institutions who completed an initial sign-up survey, 203 volunteers (157 women) completed the online experimental study. One volunteer was excluded as an outlier, taking longer than *M* + 3 *SD* in time to complete the study, giving a final sample of 202 (156 women). Participants’ ages ranged from 18 to 57 years, with a mean of 24.41 (*SD* = 7.22). The majority (*n* = 178) were students, and non-UK nationals (*n* = 116). The experimental subsample (*n* = 202) did not differ significantly on any observed variable from those who did not participate (*n* = 71). Participants were paid a fixed rate (£2.00), plus a variable amount ranging from £0.00 - £5.00 in Amazon.co.uk e-credit, dependent on their decisions in the games. The sample used in Experiment 2 was significantly younger, *t*(500.33) = −3.49, *M*_diff_ = −2.57, *p* < .001, *d* = −0.31, and consisted of more women, *χ*^2^(1) = 12.22, *p* < .001, φ = .15, more students, *χ*^2^(1) = 27.25, *p* < .001, φ = .23, and non-UK nationals, *χ*^2^(1) = 73.88, *p* < .001, φ = .38, than that of Experiment 1.

#### Materials

The same materials as described in Experiment 1 were used, but the assignment process to the economic games differed (see Procedure).

#### Measures

The same measures as described in Experiment 1 were used, with the addition of two questions at the end of the study. First, participants responded to “We are interested in the strategy (if any) you adopted when playing the economic games. Please tell us whether you were PLAYER 1 or PLAYER 2” with *player 1* / *player 2* / *unsure*. Second, participants were asked an optional free-text question: “In your own words, can you please tell us briefly why you chose to make the decisions you did in the three economic games…if you feel unable to explain your choices, please write “N/A” in the box below”.

Internal consistencies of the cognitive (α = 0.88) and affective (α = 0.81) subscales of the QCAE [9] were good. The trust measure (α = 0.77) also showed adequate internal reliability.

#### Procedure

Data were collected from two different institutions. Ethical approval was granted from the University of Sheffield’s Research Ethics Committee (project 003334), and from the Research Ethics Committees of Middlesex University (project ST002), and University College London (Chair’s approval) prior to data collection. Written informed consent was obtained from participants. The same procedure as described in Experiment 1 was followed at each institution, with the following adjustments. During sign-up, participants also completed the trust measure (rather than at the end). In the instructions, participants were ostensibly randomly assigned to be “player 1” or “player 2” *prior to* (not after) playing the economic games, and told that to determine payments, their responses would be sequentially (not randomly) matched with the next participant who was assigned as the opposite player. Participants had to click a button to be ostensibly randomly assigned as “player 1” or “player 2” by a computer algorithm (with a simulated 5 seconds delay that said “assigning participant… please wait”), before playing the economic games. All other aspects of the procedure remained the same, and participants were always assigned to be “player 1”. Study payments were calculated using the participant’s choices as player 1 and the responses of the next participant as player 2 for the IG.

### Data analysis

To maximise statistical power and obtain more accurate and stable effect estimates, data from the two experiments were combined for a fixed effects integrative data analysis [20–21]. In all regressions, experiment membership was included as a dummy variable to account for any unobserved differences between the experiments. This statistical approach also has the advantage of providing a stronger test of replication [21]. At each stage of our regression models, we tested (simultaneously) for significant interactions between experiment membership and each of the primary predictor variables (and their products). If the interaction was not significant (at α = .05), the size of the effect for that predictor between the two studies did not differ statistically, and thus the size of the effect replicated across the two experiments. In contrast, significant interactions suggest a failure to replicate across the two experiments [21].

The combined sample of 519 staff and students (353 women) had ages ranging from 18 to **77** years, with a mean of 25.97 (*SD* = 8.74), the majority (*n* = 392) were students, and UK nationals (*n* = 339). Data were analysed in R 3.2.2 [26] using packages arm [27], betareg [28], ordinal [29], and psych [30]. For the primary analyses, as DGgive, TDGgive, and IGgive were discrete ordinal outcome variables (integers between 0 and 10), and not all error distributions approximated normal, hierarchical ordered logistic regressions (proportional odds models) were used. Hierarchical models were used to estimate the main effects of the variables of interest (i.e., conditional assignment, empathy, and trust) at step 1, from their subsequent higher-order interaction terms in steps 2 and 3. As per methods used in the repeated triadic design [18], we modelled each economic decision independently, but conditioned IGgive on amounts given in the TDG to separate trust-based decisions from other-regarding behaviour, and IGexpect to account for the expected return in the IG. There was a single missing value on IGexpect in Experiment 2, which was imputed at the mean, calculated from a subset of participants from the same condition and the same value of IGgive.

For IGreciprocate, which was a continuous (294 observed variations), interval-level proportional outcome variable, a hierarchical beta regression was used, using the formula cited by Smithson and Verkuilen [31] to convert the proportion data from [0, 1] to (0, 1). IGreciprocate responses were conditioned on amount given in the DG, to assess reciprocity over and above other-regarding behaviour [18]. The advantages of the beta distribution over a standard Gaussian approach when the data are not conditionally normally distributed are detailed by Smithson and Verkuilen [31]. The results were qualitatively similar using standard OLS (Gaussian) models. To allow for inter-variable comparisons in the sizes of effects, all continuous predictors were centred and divided by 2 *SD* to be on the same scale as binary variables [32]. As all models used a logistic link, estimates are presented as odds ratios.

## Results

### Differences between the experiments

Participants in Experiment 2 (*M* = 8.45, *SD* = 1.54) did not significantly differ from those in Experiment 1 (*M* = 8.46, *SD* = 1.52) in their in-group trust for people at their institution, *t*(425.08) = −0.05, *M*_diff_ = 0.01, 95% CI [−0.28, 0.26], *p* = .960, *d* = 0.00. However, they had significantly higher levels of cognitive (*M* = 59.10, *SD* = 7.79 vs. *M* = 57.31, *SD* = 7.94), *t*(434.49) = 2.54, *M*_diff_ = 1.79, 95% CI [0.41, 3.18], *p* = .011, *d* = 0.23, and affective (*M* = 34.70, *SD* = 5.65 vs. *M* = 33.34, *SD* = 5.35), *t*(411.1) = 2.73, *M*_diff_ = 1.36, 95% CI [0.38, 2.34], *p* = .007, *d* = 0.25, empathy than Experiment 1 participants.

Table 1 shows the descriptives of the five economic variables by condition. Overall, participants in Experiment 2 gave similar amounts on average in the DG, *t*(381.23) = −0.77, *M*_diff_ = −0.16, 95% CI [−0.58, 0.25], *p* = .440, *d* = −0.07, and the TDG, *t*(432.23) = 0.12, *M*_diff_ = 0.03, 95% CI [−0.42, 0.47], *p* = .908, *d* = 0.01, and also reciprocated similar proportions in the IG, *t*(387.21) = 1.34, *M*_diff_ = .03, 95% CI [−.01, .06], *p* = .180, *d* = 0.12, to those in Experiment 1. However, Experiment 2 participants invested significantly less in the IG, *t*(423.04) = −3.14, *M*_diff_ = −0.84, 95% CI [−1.37, −0.31], *p* = .002, *d* = −0.28, yet expected higher returns, *t*(352.69) = 2.11, *M*_diff_ = .05, 95% CI [.00, .10], *p* = .036, *d* = 0.19.

**Table 1:** Descriptive statistics of economic decisions by experimental condition.

	Experiment 1 (*n* = 317)		Experiment 2 (*n* = 202)		Combined (*n* = 519)		
	Neutral	Compassion	Neutral	Compassion	Neutral	Compassion	Overall
Measure	$M_{\hat{p}}$ (*SD*)	$M_{\hat{p}}$ (*SD*)	$M_{\hat{p}}$ (*SD*)	$M_{\hat{p}}$ (*SD*)	$M_{\hat{p}}$ (*SD*)	$M_{\hat{p}}$ (*SD*)	$M_{\hat{p}}$ (*SD*)
DG_give_	.39 (.22)	.40 (.21)	.35 (.23)	.41 (.26)	.38 (.22)	.40 (.23)	.39 (.23)
TDG_give_	.32 (.25)	.37 (.26)	.30 (.24)	.39 (.25)	.32 (.24)	.38 (.26)	.35 (.25)
IG_give_	.61 (.30)	.61 (.29)	.47 (.31)	.57 (.28)	.56 (.31)	.59 (.29)	.58 (.30)
IG_expect_^1^	.42 (.22)	.39 (.22)	.46 (.32)	.46 (.25)	.44 (.26)	.42 (.24)	.43 (.25)
IG_reciprocate_	.40 (.18)	.41 (.20)	.40 (.23)	.45 (.21)	.40 (.20)	.43 (.21)	.41 (.20)

$M_{\hat{p}}$ = mean proportion.

^1^Two values in Experiment 1 and eleven values in Experiment 2 recoded as the maximum possible expected return, as actual value provided was higher than possible.

### Assignment and manipulation checks

The experimental (*n* = 260) and control (*n* = 259) groups did not differ significantly on observed characteristics measured prior to assignment (Welch’s *t*-tests). Levels of cognitive, *t*(515.63) = 0.44, *M*_diff_ = 0.31, 95% CI [−1.06, 1.68], *p* = .658, *d* = 0.04, and affective, *t*(500.07) = 0.36, *M*_diff_ = 0.17, 95% CI [−0.78, 1.12], *p* = .719, *d* = 0.03, empathy did not differ significantly across the groups. Furthermore, self-reported trust did not differ by assignment, *t*(516.92) = 0.04, *M*_diff_ = 0.01, 95% CI [−0.26, 0.27], *p* = .965, *d* = 0.00.

The experimental images produced a significantly different affective profile in participants than the control images. Differences were observed in all five affective states measured (see S3 Text), including higher levels of compassion (*M* = 3.23, *SD* = 0.72) in the experimental than the neutral condition (*M* = 2.21, *SD* = 0.86), *t*(500.88) = 14.66, *M*_diff_ = 1.02, 95% CI [0.89, 1.16], *p* < .001, *d* = 1.29; higher levels of sadness in the experimental (*M* = 2.89, *SD* = 0.78) than neutral (*M* = 1.52, *SD* = 0.67) condition, *t*(506.15) = 21.54, *M*_diff_ = 1.37, 95% CI [1.24, 1.49], *p* < .001, *d* = 1.89; and higher levels of disgust in the experimental (*M* = 2.27, *SD* = 0.90) than neutral (*M* = 1.13, *SD* = 0.42) condition, *t*(364.84) = 18.51, *M*_diff_ = 1.14, 95% CI [1.02, 1.26], *p* < .001, *d* = 1.62. Compassion was the dominant emotion in the experimental condition, followed by sadness (*M* = 2.89, *SD* = 0.78), *t*(259) = 6.68, *Mdiff* = 0.35, 95% CI [0.24, 0.45], *p* < .001, *d* = 0.46. The dominant emotion in the control condition was happiness (*M* = 2.79, *SD* = 0.61), followed by compassion (*M* = 2.21, *SD* = 0.86), *t*(258) = 11.05, *M*_diff_ = 0.58, 95% CI [0.47, 0.68], *p* < .001, *d* = 0.77. Supplementary tests (see S3 Text) indicated that responses in the economic tasks did not appear to be driven by variance in any one of the five affective states measured in particular.

The mean memory score was significantly higher in the neutral (*M* = 4.19, *SD* = 0.86) than experimental (*M* = 4.03, *SD* = 0.79) condition, *t*(512.94) = −2.13, *M*_diff_ = −0.15, 95% CI [−0.30, −0.01], *p* = .034, *d* = −0.19. In Experiment 2, 194 out of 202 (96%) participants correctly reported that they were “player 1”, with 6 (3%) answering “player 2” and 2 (1%) “unsure”, suggesting effective assignment.

### Economic decisions

Offers in the DG were somewhat higher than that typically reported (e.g., 21% [19]; 25% [18]), yet lower than some (e.g., 59% [10]). A meta-analysis [33] reported that dictators, on average, give 28.35%, with a positively skewed distribution. There was a greater tendency in our sample for participants to give 50% of their endowment in the DG (50% was the modal choice, with 260 participants giving half).

While the TDG has not been studied widely, our results are consistent with Cox [19] who found participants sent approximately 36% of their fund, yet are higher than Ashraf et al. [18] who reported a mean of 24%. We observe similar consistency for the IG, where Cox [19] found participants invested 60% on average. A meta-analysis of the trust game [34] reported that participants, on average, invest 50% of their endowment and return 37%. Ashraf et al. [18] recorded lower levels of investment (45%) and reciprocity (27%). The three games elicited different decision processes. There is clear evidence of self-invested interest in these data; participants gave significantly more in the IG than in the TDG, *t*(518) = 16.13, *M*_diff_ = 2.29, 95% CI [2.01, 2.57], *p* < .001, *d* = 0.83. There is also evidence for reciprocity; the average proportion returned in the IG was significantly higher than offers in the DG, *t*(518) = 2.02, *M*_diff_ = 0.02, 95% CI [0.00, 0.05], *p* = .044, *d* = 0.11. Participants gave significantly less in the TDG than the DG (see [19]), *t*(518) = −3.79, *M*_diff_ = −0.45, 95% CI [−0.68, −0.22], *p* < .001, *d* = −0.19.

Participants gave statistically similar amounts in the DG, *t*(515.91) = 1.36, *M*_diff_ = 0.27, 95% CI [−0.12, 0.66], *p* = .174, *d* = 0.12, and IG, *t*(512.46) = 1.49, *M*_diff_ = 0.39, 95% CI [−0.13, 0.91], *p* = .138, *d* = 0.13, and expected similar amounts in return in the IG, *t*(510.12) = −0.88, *M*_diff_ = −0.02, 95% CI [−0.06, 0.02], *p* = .379, *d* = −0.08, across the two conditions. However, those in the experimental condition gave significantly more in the TDG than those in the neutral condition, *t*(515.94) = 2.83, *M*_diff_ = 0.62, 95% CI [0.19, 1.05], *p* = .005, *d* = 0.25, and there was also a trend for increased reciprocation in the experimental condition, *t*(516.68) = 1.79, *M*_diff_ = 0.03, 95% CI [0.00, 0.07], *p* = .074, *d* = 0.16. Full distributions of the outcome variables are in S1 Fig.

### Hierarchical regressions

Table 2 shows results of the ordered logit models for amounts given in the DG and TDG and Table 3 shows the results of the ordered logit and beta model for amount given and reciprocated in the IG. Step one included all control variables, trust, empathy, and condition. In step two, we interacted the dispositional empathy and trust variables with condition, and in step three modelled the three-way interactions between these variables.

**Table 2:** Hierarchical ordered logistic regressions predicting giving in the DG and TDG.

	DGgive				TDGgive			
		95% CI				95% CI		
Predictor	OR	LO	HI	*p*	OR	LO	HI	*p*
**Step 1**	**LLV = −842.78**				**LLV = −1017.3**			
	**χ**^**2**^**(13) = 25.01, *p* = .023**				**χ**^**2**^**(13) = 35.11, *p* = .001**			
Experiment (0 = Experiment 1)	0.93	0.64	1.35	.699	1.07	0.75	1.53	.694
TDG before DG	1.12	0.79	1.58	.526	-	-	-	-
IG before DG	0.82	0.58	1.16	.266	-	-	-	-
DG before TDG	-	-	-	-	0.98	0.71	1.35	.885
IG before TDG	-	-	-	-	0.78^a^	0.56	1.08	.130
Gender (0 = male)	0.96	0.66	1.39	.828	0.79	0.55	1.14	.204
Age	1.05	0.70	1.60	.811	0.89	0.59	1.34	.584
Nationality (0 = UK)	0.71	0.49	1.03	.067	0.85	0.59	1.22	.375
Affiliation (0 = student)	1.34	0.81	2.23	.250	1.29	0.80	2.09	.301
Economics (0 = no)	0.43	0.14	1.34	.136	0.26	0.09	0.79	.016
Time taken	1.30	0.95	1.80	.108	0.92	0.67	1.24	.615
Trust	1.37	0.99	1.89	.057	1.59	1.17	2.16	.003
CE	1.32	0.94	1.86	.110	1.29	0.93	1.79	.128
AE	1.13	0.79	1.62	.506	1.08	0.77	1.51	.668
Condition (0 = neutral)	1.25	0.90	1.73	.182	1.67	1.22	2.27	.001
**Step 2**	**χ**^**2**^**(5) = 5.90, *p* = .316**				**χ**^**2**^**(5) = 7.89, *p* = .162**			
CE:Condition	1.57	0.78	3.14	.203	1.11	0.57	2.15	.764
AE:Condition	0.90	0.45	1.81	.768	1.54^b^	0.80	2.97	.200
Trust:Condition	0.59	0.30	1.14	.118	0.48	0.26	0.90	.022
CE:Trust	0.94	0.51	1.74	.853	1.14	0.66	2.02	.637
AE:Trust	1.47	0.80	2.70	.209	1.23	0.71	2.13	.461
**Step 3**	**χ**^**2**^**(2) = 9.13, *p* = .010**				**χ**^**2**^**(2) = 0.07, *p* = .967**			
CE:Trust:Condition	1.40^a^	0.40	4.79	.596	1.11	0.32	3.68	.869
AE:Trust:Condition	0.15	0.04	0.53	.003	0.86	0.27	2.76	.802

*N* = 519. Continuous predictors rescaled by centring and dividing by 2 *SD* to put them on the same scale as binary variables [27]. Odds ratios and CIs calculated by the exponentiation of log estimates, inferential tests conducted on the log scale. CE, cognitive empathy; AE, affective empathy; DG, dictator game; TDG, triple dictator game; IG, investment game.

^a^Estimates differed significantly (*p* < .05) across the two experiments.

^b^Estimates borderline differed significantly (*p* < .10) across the two experiments.

**Table 3:** Hierarchical ordered logistic and beta regressions predicting giving and reciprocating in the IG.

	IGgive				IGreciprocate			
		95% CI				95% CI		
Predictor	OR	LO	HI	*p*	OR	LO	HI	*p*
**Step 1**	**LLV = −992.64**				**LLV = 35.12**			
	**χ**^**2**^**(15) = 127.6, *p* < .001**				**χ**^**2**^**(14) = 49.38, *p* < .001**			
Experiment (0 = Experiment 1)	0.68	0.47	0.97	.033	1.06	0.86	1.31	.559
DGgive	-	-	-	-	1.72	1.43	2.07	.000
TDGgive	3.80	2.65	5.50	.000	-	-	-	-
IGexpect^1^	1.91^a^	1.36	2.71	.000	-	-	-	-
DG before IG	0.84	0.60	1.18	.315	0.92^a^	0.76	1.11	.371
TDG before IG	1.15	0.82	1.60	.418	1.05	0.87	1.27	.633
Gender (0 = male)	0.53	0.36	0.77	.001	0.96^a^	0.78	1.18	.683
Age	1.14	0.76	1.74	.528	1.08	0.86	1.36	.495
Nationality (0 = UK)	0.49	0.34	0.70	.000	1.13	0.92	1.39	.252
Affiliation (0 = student)	0.64	0.39	1.04	.071	0.96	0.73	1.26	.772
Economics (0 = no)	1.38	0.47	4.26	.567	0.70	0.38	1.31	.268
Time taken	1.52	1.07	2.36	.038	0.99	0.83	1.18	.895
Trust	1.30	0.95	1.78	.099	1.15	0.96	1.38	.125
CE	0.75	0.54	1.05	.099	1.09	0.90	1.32	.363
AE	1.14	0.80	1.61	.479	1.05	0.86	1.28	.632
Condition (0 = neutral)	1.19^b^	0.87	1.64	.276	1.15	0.96	1.38	.129
**Step 2**	**χ**^**2**^**(5) = 10.71, *p* = .058**				**χ**^**2**^**(5) = 3.60, *p* = .609**			
CE:Condition	1.99	1.00	3.97	.050	0.89	0.61	1.31	.564
AE:Condition	1.37	0.69	2.73	.368	1.12	0.77	1.65	.549
Trust:Condition	0.78	0.41	1.46	.435	1.22	0.84	1.76	.299
CE:Trust	0.92	0.51	1.66	.791	1.21	0.87	1.69	.257
AE:Trust	1.72	0.97	3.07	.064	0.83^a^	0.59	1.15	.257
**Step 3**	**χ**^**2**^**(2) = 7.86, *p* = .020**				**χ**^**2**^**(2) = 7.77, *p* = .021**			
CE:Trust:Condition	0.18	0.06	0.60	.005	0.80^b^	0.40	1.60	.530
AE:Trust:Condition	1.91	0.58	6.41	.289	2.75^a^	1.35	5.59	.005

*N* = 519. Continuous predictors rescaled by centring and dividing by 2 *SD* to put them on the same scale as binary variables [27]. Odds ratios and CIs calculated by the exponentiation of log estimates, inferential tests conducted on the log scale. CE, cognitive empathy; AE, affective empathy; DG, dictator game; TDG, triple dictator game; IG, investment game.

^1^Thirteen values recoded as the maximum possible expected return, as value provided was higher than possible.

^a^Estimates differed significantly (*p* < .05) across the two experiments.

^b^Estimates borderline differed significantly (*p* < .10) across the two experiments.

#### Consistent effects

Condition was a significant positive predictor of TDGgive, OR = 1.67, 95% CI [1.22, 2.27], *p* = .001, with participants giving more in the experimental condition, but was not a significant predictor of any other economic outcomes at the aggregate level. Trust was a borderline significant predictor of DGgive, OR = 1.37, 95% CI [0.99, 1.89], *p* = .057, a significant predictor of TDGgive, OR = 1.59, 95% CI [1.17, 2.16], *p* = .003, and had a non-significant trend for predicting IGgive, OR = 1.30, 95% CI [0.95, 1.78], *p* = .099, when conditioned on other variables. No significant main effects of empathy were observed, but there was a non-significant trend for cognitive empathy to predict less IGgive, OR = 0.75, 95% CI [0.54, 1.05], *p* = .099.

The absence of main effects was qualified by significant interaction terms. The three-way interaction between affective empathy, trust, and condition significantly predicted DGgive, OR = 0.15, 95% CI [0.04, 0.53], *p* = .003. The product of trust and condition significantly predicted TDGgive, OR = 0.48, 95% CI [0.26, 0.90], *p* = .022. The product of cognitive empathy and condition significantly predicted IGgive, OR = 1.99, 95% CI [1.00, 3.97], *p* = .050 (.04998, when expanded to the fifth decimal place), while the three-way interaction between cognitive empathy, trust, and condition significantly did so, OR = 0.18, 95% CI [0.06, 0.60], *p* = .005.

#### Inconsistent effects

The effect of the three-way interaction between cognitive empathy, trust, and condition predicting DGgive differed significantly (*p* < .05) across experiments, with estimates of OR = 0.27, 95% CI [0.05, 1.35], *p* = .112 when experiment = 1 and OR = 20.86, 95% CI [2.45, 188.35], *p* = .006 when experiment = 2. The product of affective empathy and condition predicting TDGgive had nearly significantly different (*p* = .05) effects across the two studies, with estimates of OR = 2.56, 95% CI [1.09, 6.05], *p* = .031 when experiment = 1 and OR = 0.64, 95% CI [0.21, 1.90], *p* = .419 when experiment = 2. Experimental condition had borderline significantly different (*p* = .068) effects across the two studies, with estimates of OR = 0.97, 95% CI [0.65, 1.44], *p* = .869 when experiment = 1 and OR = 1.70, 95% CI [1.00, 2.88], *p* = .049 when experiment = 2. The product of affective empathy and trust had significantly different effects across experiments when predicting IGreciprocate, with estimates of OR = 1.24, 95% CI [0.80, 1.94], *p* = .334 when experiment = 1 and OR = 0.48, 95% CI [0.28, 0.83], *p* = .009 when experiment = 2. Finally, the estimates of the three-way interaction between affective empathy, trust, and condition predicting IGreciprocate differed significantly across the two experiments, with estimates of OR = 0.91, 95% CI [0.41, 2.03], *p* = .817 when experiment = 1 and OR = 10.31, 95% CI [3.10, 34.26], *p* < .001 when experiment = 2.

#### Simple effects

**Fig 1:**
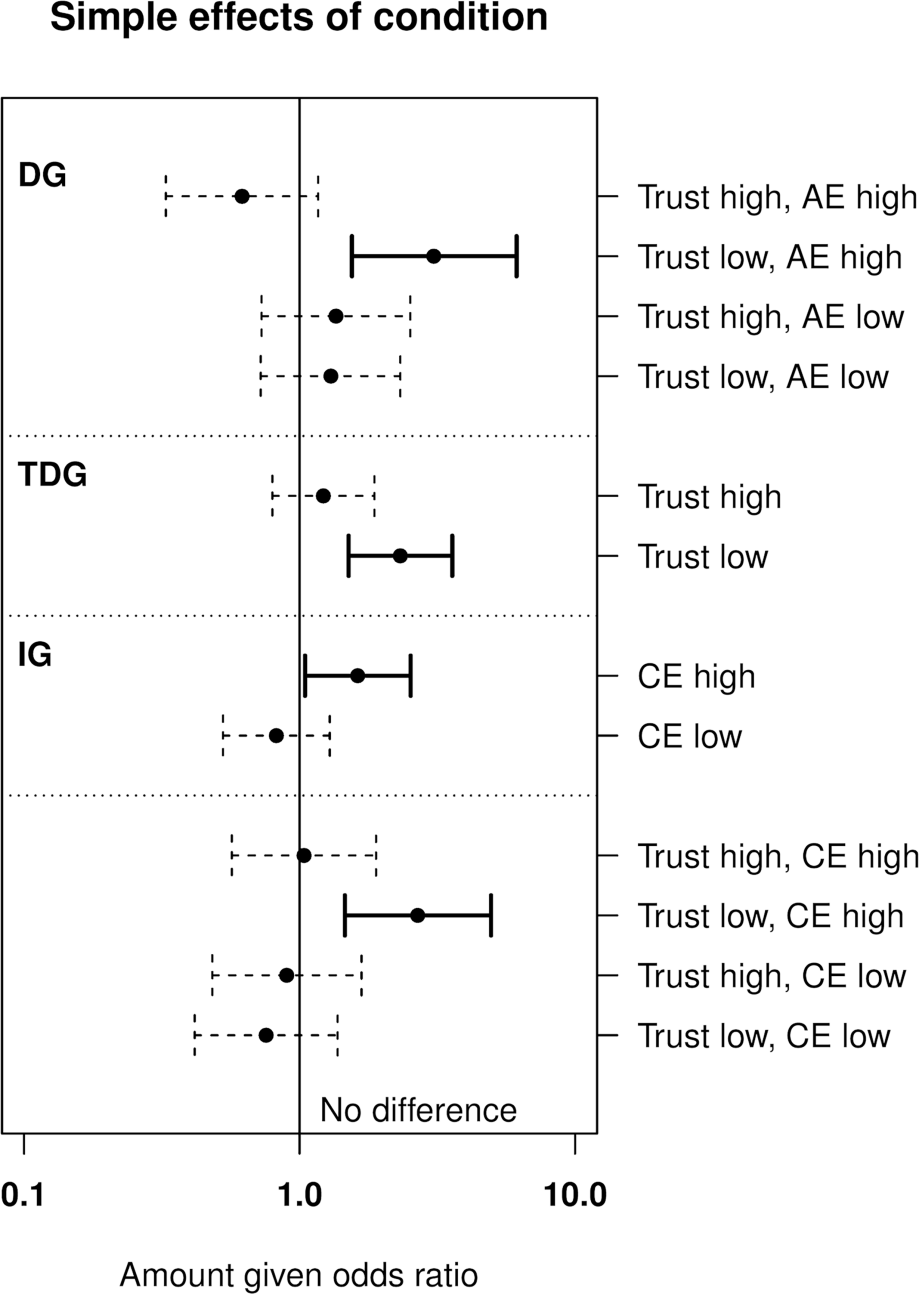
Simple effects of experimental condition on amount given in the economic games for consistent (replicated) interactions. Effect of condition (0 = neutral, 1 = experimental) estimated at high (+ 1 SD) and low (− 1 SD) levels of moderator variables. Odds ratios and CIs calculated by the exponentiation of log estimates, inferential tests conducted on the log scale, x-axis is on the log scale. AE, affective empathy; CE, cognitive empathy; DG, dictator game; IG, investment game; TDG, triple dictator game. Error bars show 95% CIs. Estimates with solid bold CIs are significant at *p* < .05.

Fig 1 shows simple effects for significant (*p* < .05) and nearly significant (*p* = .05) interactions that had consistent estimates across both experiments, as the effect of condition at +/− 1 *SD* of moderators. These simple effects were constructed by removing all covariate terms (except for the “experiment” dummy and TDGgive and IGexpect for the IGgive model, as described in the data analysis), and modelling each interaction individually (with only the requisite lower-order terms included). Simple slopes for condition were then estimated and tested, as described by Aiken and West [35].

Condition had a significant positive effect on DGgive when trust was low and affective empathy was high, OR = 3.07, 95% CI [1.55, 6.14], *p* = .001. Condition had a significant positive effect on TDGgive when trust was low, OR = 2.32, 95% CI [1.51, 3.58], *p* < .001. Condition had a significant positive effect on IGgive when cognitive empathy was high, OR = 1.63, 95% CI [1.05, 2.53], *p* = .031. Finally, condition had a significant positive effect on IGgive when trust was low and cognitive empathy was high, OR = 2.68, 95% CI [1.46, 4.95], *p* = .002. No other simple effects were statistically significant.

### Qualitative data

A thematic analysis was conducted on responses to the optional free-text question in Experiment 2. As the majority of responses discussed the three games collectively, responses were collapsed. Six themes were derived from these data:

1. “Equality/fairness”: participants described wanting to achieve equality or fairness, or sharing their money with others.
2. “Maximising own gain”: participants described wanting to maximise their own monetary or psychological benefit(s), including winning the game(s), or maintaining a sense of power.
3. “Reciprocity”: participants described decisions contingent on the other player’s positive actions, such as expecting the other person to act in their favour, or trusting the other player to return money.
4. “Risk averse”: participants described not wanting to take risks, or not trusting the other player. This may include expectations of negative reciprocity from the other player.
5. “Generosity”: participants described being generous, or selfless, or wanting to be generous/selfless towards others, or state that the other player would benefit from the money more than they would.
6. “No strategy/instinct”: participants described having no clear strategy in their decisions, or that they were random, or that they were following their instinct.

Two independent coders were provided with these themes and definitions and asked to code each response (for whether a theme was present [1] or absent [0]). Cohen’s κ was calculated to determine interrater agreement with our initial coding, and ranged from 0.76 (substantial agreement) to 0.95 (almost perfect agreement).

Table 4 shows the frequency of participants endorsing each theme by condition. Participants in the experimental condition were qualitatively more likely to endorse themes of equality/fairness and generosity, and less likely to endorse maximising their own gain and having no strategy. However, these differences were not statistically significant. Participants in the experimental condition were significantly more likely to report decisions contingent on expectations of the other player’s positive actions or reciprocity (*p* = .031), and significantly less likely to report being risk averse, or *not trusting* the other player (*p* = .009).

**Table 4:** Themes derived from the qualitative data (Experiment 2).

Strategy	Control	Experimental	% reliability	Cohen’s kappa	Fisher’s exact *p*
Equality/fairness	42	54	94.03	.88	.290
Maximising own gain	27	24	92.90	.82	.409
Reciprocity	27	45	90.34	.80	.031
Risk Averse	16	5	94.60	.76	.009
Generosity	7	15	95.74	.81	.170
No strategy/instinct	7	5	99.43	.95	.554
Total responses	84	92	94.51	.84	-

*N* = 176. Items coded for presence (1) or absence (0) of theme. Total responses are less than sum of codings as categories were not mutually exclusive. Reliability statistics averaged over two independent coders.

## Discussion

Past research has suggested a link between exposure to themes of suffering and vulnerability and altruistic economic behaviour. However, there has been little investigation into the effects of such exposure on other economic decisions, such as those involving trust and reciprocity, and the roles of dispositional empathy and self-reported trust in this process have typically been overlooked. Using the largest experimental sample to date to explore this issue, we observed a significant aggregate main effect of exposure to imagery depicting suffering and vulnerability on altruistic giving in the TDG. However, we failed to replicate studies that have shown an effect on altruistic giving in the standard DG [6, 10, 14], highlighting key differences between the two tasks. This was an unexpected finding, as the two games are both designed to measure altruistic responding, but may be explained by key structural differences between them. First, on average, participants (across both conditions) played the two games differently, giving a statistically meaningful lower amount in the TDG than the DG, demonstrating that participants take the overall resulting distribution of wealth between the two players (and not just the amount “donated”) into account. Second, in our version of the DG fairness dominated [36], with the majority of participants choosing to opt for a 50/50 split (not possible in the TDG) regardless of experimental assignment, suggesting that distributional fairness norms may have been more powerful than the experimental manipulation in this context [37]. In the TDG, one must make a decision about whether they or another person will receive a greater amount overall. The TDG can thus potentially be considered a more sensitive measure of altruistic decision-making than the DG and this presents a methodological consideration for those designing future work in this area.

We failed to observe any significant main effects of condition on trust-based or reciprocated decisions, as measured by the IG, suggesting that the decision-making context (i.e., altruistic or self-interested) was a determinant of the effects of the experimental manipulation, at the aggregate level. Contrary to predictions, we also found no main effects of participants’ dispositional empathy on giving (cf., [12]), except for a trend for those with greater cognitive empathy to give less in the investment game. Self-reported trust for the recipient did positively predict giving in all three games, however.

Moving beyond the aggregate level of analysis, we observed substantial heterogeneity in the effects of the experimental manipulation on giving in all three economic games. In particular, the manipulation was most effective at increasing monetary giving in a subset of participants who reported lower levels of trust for their recipient. In the TDG, the positive effect of the experimental manipulation was concentrated in those lower rather than higher in trust. In the DG, the only positive effect of condition occurred when trust was low and affective empathy was high. In the IG, a positive effect of condition occurred when trust was low and cognitive empathy was high. This pattern of findings, alongside complementary qualitative evidence of greater expectations of positive actions from the other player and less risk/greater expected reciprocation in those in the experimental condition, suggests that the experimental manipulation may have compensated for the effects of lower levels of trust on giving in both altruistic *and* trust-based economic decisions. Indeed, as in previous studies [5], it appears that the pictorial experimental manipulation was most effective in those participants that were otherwise giving less. This finding extends past work to suggest a *potential* affiliative effect of exposure to stimuli depicting suffering and vulnerability on economic trust-based decisions towards strangers, for certain people. Of particular interest is the apparent inconsistency between self-reported trust that was equivalent across conditions in Experiment 1, and increased implicit trust in the experimental condition that is evident in the qualitative data in Experiment 2. One potential explanation is that the trust measure unaffected by experimental assignment in Experiment 1 is a trait measure of trust for the institutional in-group, while that indexed by the qualitative data is more of a state, or “in-the-moment”, measure of trust or expected reciprocation on the economic task, which one would expect to be more malleable. Thus, the two findings are reconcilable.

The differential moderating effects of affective and cognitive empathy in the DG and IG, respectively, support and extend prior work, whereby, upon exposure to emotional content, affective empathy has been found to be more important in predicting altruistic decisions [10], and cognitive empathy may be more likely to be utilised in strategic, self-invested decisions [22]. For example, those with higher cognitive empathy (and lower trust) may have expected their recipients (ostensibly exposed to the same stimuli as them) to return more in the experimental (vs. neutral) condition. This interpretation is consistent with the qualitative findings from Experiment 2. Indeed, while cognitive empathy was negatively correlated with expected returns in the neutral condition, *r* = −.15, 95% CI [−.27, −.03], *p* = .017, it was positively correlated with expected returns in the experimental condition, *r* = .10, 95% CI [−.02, .22], *p* = .109. These correlations differ significantly, *z* = 2.85, *p* = .004. Note however that main effects of dispositional (affective and cognitive) empathy on economic behaviour were not observed (cf., [12]), yet altruistic motivations are only one potential source of prosocial behaviour, and other factors may be involved [38].

No consistent effects of trust, empathy, or condition (or their products) were observed in reciprocated decisions. While this result contrasts some prior work, showing, for example, increased trustworthiness (reciprocity) as a function of self-reported trust [18], it is consistent with research suggesting that compassion training primarily affects altruistically-motivated, rather than reciprocity-based, helping behaviour [39]. Two points are worth mentioning, however. First, as with the DG, there was some evidence that affective empathy and trust moderated the effects of condition on reciprocated decisions in Experiment 2 (but not in Experiment 1, see below). Second, this was the only economic decision measured using the strategy method; there is evidence that the strategy method reveals lower trustworthiness than actual gameplay in the IG [40] (but cf. [18]).

Finally, as well as the consistent effects discussed above, several effects differed significantly between the two experiments, suggesting that they were the either the result of methodological artefacts, or systematic unmodelled differences between the two experimental paradigms. While we refrain from drawing any conclusions from these effects at this stage, they remain to be evaluated against the results from future studies, and are otherwise largely consistent with the pattern of findings discussed above.

We have several limitations to acknowledge. First, the studies were conducted in university institutions with staff and students, while our effects held across participants from three independent institutions, with different demographic compositions; we cannot infer that the results would necessarily hold for people in other settings. Second, while decision-making was incentivized, the effects are grounded in the size of the incentives used, and may not generalize when the amounts of money involved are altered. It is also worth reflecting that participants were not paid instantly as a result of their decisions, but were paid some weeks later, potentially diminishing the value of the reward. Third, no alpha correction was applied, as procedures such as the Bonferroni correction render analyses extremely conservative as a function of the number of tests reported [41]. Instead, readers may apply post-hoc corrections to the alpha values, if they wish to. In particular, given the number of tests and lack of specific directional hypotheses, the results of the interactions in this study should be considered exploratory, and warrant confirmation in further studies. Fourth, while exposure to the experimental images produced changes in the participants’ affective profiles, supplementary analyses indicated that it was not possible in these data to identify a specific causal mechanism (e.g., increased compassion) for the results observed. Fifth, while behaviour differed across the economic games that suggested strategic gameplay, no data were collected to confirm that participants believed they were paired with other real people, that their payments would be determined based on their responses, or which condition they thought their partner was in. These factors could have affected the results observed. Sixth, the results in this study are grounded in the particular measures used (such as the QCAE; Reniers et al., 2011), which showed little explanatory power; future work may consider an exploration of alternative measures, including personality dimensions, in explaining behaviour across economic games. Finally, participants in both conditions played the economic games with the same anonymous recipients. This paradigm was designed to enable us to isolate the effects of the experimental manipulation on giving to the same target across the three games. Participants’ behaviour, of course, may differ if the target of their economic decisions is altered (i.e. to the person experiencing suffering themselves [33]).

## Conclusion

In sum, we observe a complex relationship between exposure to stimuli depicting the suffering and vulnerability of others and economic decision-making, which differs as a function of the decision-making context. Our findings reinforce the link between exposure to suffering others and average other-regarding, or altruistic, economic behaviour in one economic index (the “triple dictator game”), but not in the standard “dictator game”. Conversely, we found effects of the experimental manipulation in the dictator game and increased trust-based giving in the “investment game”, for some people only. In particular, those who reported lower trust for their recipient and, dependent on decisional motivations, for those higher in affective empathy (affect sharing) in other-regarding decisions in the dictator game, and cognitive empathy (perspective-taking) in strategic, trust-based decisions in the investment game. Attempts to utilise suffering and vulnerability primes to influence economic decision-making may thus be most effective if they are tailored, taking both contextual motivations and individual differences in trust and empathy into account.

## Supporting information

S1 Text Experimental game instructions (Experiment 1). (DOCX)

S2 Text Dataset variable definitions. (DOCX)

S3 Text Associations between affective profiles and primary economic outcomes. (DOCX)

S1 Fig Distributions (histograms) of economic outcomes. (TIF)

S1 Dataset Supporting data. (CSV)
