# Supplementary material for: The effects of exposure to images of others' suffering and vulnerability on altruistic, trust-based, and reciprocated economic decision-making
Source: PLoS One. 2018 Mar 21;13(3):e0194569. doi: 10.1371/journal.pone.0194569 (PMC5862494; doi:10.1371/journal.pone.0194569)
Supplement: S1 Text — (DOCX) [file pone.0194569.s001.docx]

**Supporting Information (S1)**

**Experimental game instructions (Experiment 1)**

You will now play three economic 'games' (A, B, and C) in a random order. These are all two-player games.

You will respond to the games on your own, but your responses will be randomly and anonymously paired with those of another study participant who is also a member of staff or a student at the [host institution].

The participant you are paired with will also be told that their responses are randomly and anonymously paired with those of another study participant (you), but you do not know who they are, and they do not know who you are.

Each game uses 'experimental currency units'. Each currency unit has a value of 10p (i.e., 10 CUs = £1.00).

*Remember: 1 currency unit = 10p; 10 currency units = £1.00.*

**Game A** has the following rules:

i) Player 1 starts with 10 currency units. Player 2 starts with 0 currency units.

ii) Player 1 can give an amount (0 - 10) of their currency units to Player 2. Player 1 keeps the rest.

The following table illustrates how the game works for each possible decision.

| **STAGE 1: Player 1 gives an amount to Player 2.** | **STAGE 2: Player 2 receives amount from Player 1.** |
| --- | --- |
| Player 1 gives 0, keeps 10 | Player 2 receives 0 |
| Player 1 gives 1, keeps 9 | Player 2 receives 1 |
| Player 1 gives 2, keeps 8 | Player 2 receives 2 |
| Player 1 gives 3, keeps 7 | Player 2 receives 3 |
| Player 1 gives 4, keeps 6 | Player 2 receives 4 |
| Player 1 gives 5, keeps 5 | Player 2 receives 5 |
| Player 1 gives 6, keeps 4 | Player 2 receives 6 |
| Player 1 gives 7, keeps 3 | Player 2 receives 7 |
| Player 1 gives 8, keeps 2 | Player 2 receives 8 |
| Player 1 gives 9, keeps 1 | Player 2 receives 9 |
| Player 1 gives 10, keeps 0 | Player 2 receives 10 |

**The total income of Player 1 is the amount they keep in STAGE 1.**

**The total income of Player 2 is the amount received in STAGE 2.**

**You are Player 1.** How many of your 10 currency units would you like to give to Player 2?


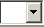


*Remember: 1 currency unit = 10p; 10 currency units = £1.00*

**Game B** has the following rules:

i) Player 1 starts with 10 currency units. Player 2 starts with 0 currency units.

ii) Player 1 can give an amount (0 - 10) of their currency units to Player 2. Player 1 keeps the rest.

iii) The amount Player 1 chooses to give to Player 2 is tripled by the experimenter, before giving it to Player 2.

The following table illustrates how the game works for each possible decision.

| **STAGE 1: Player 1 gives an amount to the experimenter.** | **STAGE 2: Amount is tripled by experimenter and given to Player 2.** |
| --- | --- |
| Player 1 gives 0, keeps 10 | Player 2 receives 0 |
| Player 1 gives 1, keeps 9 | Player 2 receives 3 |
| Player 1 gives 2, keeps 8 | Player 2 receives 6 |
| Player 1 gives 3, keeps 7 | Player 2 receives 9 |
| Player 1 gives 4, keeps 6 | Player 2 receives 12 |
| Player 1 gives 5, keeps 5 | Player 2 receives 15 |
| Player 1 gives 6, keeps 4 | Player 2 receives 18 |
| Player 1 gives 7, keeps 3 | Player 2 receives 21 |
| Player 1 gives 8, keeps 2 | Player 2 receives 24 |
| Player 1 gives 9, keeps 1 | Player 2 receives 27 |
| Player 1 gives 10, keeps 0 | Player 2 receives 30 |

**The total income of Player 1 is the amount they keep in STAGE 1.**

**The total income of Player 2 is the amount received in STAGE 2.**

**You are Player 1.** How many of your 10 currency units would you like to give to Player 2?


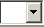


*Remember: 1 currency unit = 10p; 10 currency units = £1.00*

**Game C** has the following rules:

i) Player 1 starts with 10 currency units. Player 2 starts with 0 currency units.

ii) Player 1 can give an amount (0 - 10) of their currency units to Player 2. Player 1 keeps the rest.

iii) The amount Player 1 chooses to give to Player 2 is tripled by the experimenter, before it is given to Player 2.

iv) Player 2 has the opportunity to give an amount (0 - 30) of the tripled currency units they received back to Player 1.

The following table illustrates how the game works for each possible decision.

| **STAGE 1: Player 1 gives an amount to the experimenter.** | **STAGE 2: Amount is tripled and given to Player 2.** | **STAGE 3: Player 2 can give back an amount to Player 1.** |
| --- | --- | --- |
| Player 1 gives 0, keeps 10 | Player 2 receives 0 | Player 2 returns 0, keeps 0 |
| Player 1 gives 1, keeps 9 | Player 2 receives 3 | Player 2 returns 0-3, keeps remainder |
| Player 1 gives 2, keeps 8 | Player 2 receives 6 | Player 2 returns 0-6, keeps remainder |
| Player 1 gives 3, keeps 7 | Player 2 receives 9 | Player 2 returns 0-9, keeps remainder |
| Player 1 gives 4, keeps 6 | Player 2 receives 12 | Player 2 returns 0-12, keeps remainder |
| Player 1 gives 5, keeps 5 | Player 2 receives 15 | Player 2 returns 0-15, keeps remainder |
| Player 1 gives 6, keeps 4 | Player 2 receives 18 | Player 2 returns 0-18, keeps remainder |
| Player 1 gives 7, keeps 3 | Player 2 receives 21 | Player 2 returns 0-21, keeps remainder |
| Player 1 gives 8, keeps 2 | Player 2 receives 24 | Player 2 returns 0-24, keeps remainder |
| Player 1 gives 9, keeps 1 | Player 2 receives 27 | Player 2 returns 0-27, keeps remainder |
| Player 1 gives 10, keeps 0 | Player 2 receives 30 | Player 2 returns 0-30, keeps remainder |

**The total income of Player 1 is the amount they keep in STAGE 1, *plus* the amount returned to them in STAGE 3.**

**The** **total income of Player 2 is the amount received in STAGE 2, *minus* the amount returned to Player 1 in STAGE 3.**

**You are Player 1.** How many of your 10 currency units would you like to give to Player 2?


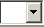


**The currency units you gave are tripled for Player 2.** How many units do you expect Player 2 to return to you?

Please enter an amount in the box below.


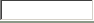


**Now assume that you are Player 2.**

For each amount of currency units that you might receive from the experimenter (which is triple the initial amount given by Player 1), indicate how many currency units you would return to Player 1.

| 3 | 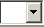 |
| --- | --- |
| 6 | 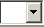 |
| 9 | 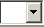 |
| 12 | 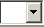 |
| 15 | 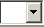 |
| 18 | 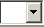 |
| 21 | 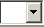 |
| 24 | 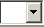 |
| 27 | 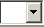 |
| 30 | 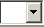 |
