# Supplementary material for: The effects of exposure to images of others' suffering and vulnerability on altruistic, trust-based, and reciprocated economic decision-making
Source: PLoS One. 2018 Mar 21;13(3):e0194569. doi: 10.1371/journal.pone.0194569 (PMC5862494; doi:10.1371/journal.pone.0194569)
Supplement: S2 Text — (DOCX) [file pone.0194569.s002.docx]

**Supporting Information (S2)**

**Dataset variable definitions**

| **Variable name** | **Definition** | **Type** | **Values** |
| --- | --- | --- | --- |
| AE | Trait affective empathy (QCAE) | Continuous | - 18-47 |
| Affiliation | Staff/student affiliation | Binary | - 0 = Student - 1 = Staff |
| Age | Participant’s age | Continuous | - 18-77 |
| CE | Trait cognition empathy (QCAE) | Continuous | - 29-76 |
| Coding#_X | Whether coder # agrees the qualitative response fits topic X (exp. 2) | Binary | - 0 = No - 1 = Yes |
| Compassion | Compassion following induction | Ordinal | - 1-4 |
| Condition | Condition membership | Binary | - 0 = Neutral - 1 = Compassion |
| DGbfIG | Order indicator of dictator game before investment game | Binary | - 0 = No - 1 = Yes |
| DGbfTDG | Order indicator of dictator game before triple dictator game | Binary | - 0 = No - 1 = Yes |
| DGgive | Amount given in the dictator game | Ordinal | - 0-10 |
| Disgust | Disgust following induction | Ordinal | - 1-4 |
| Econ | Affiliation with economics | Binary | - 0 = No - 1 = Yes |
| Experiment | Experimental membership | Binary | - 0 = Experiment 1 - 1 = Experiment 2 |
| Gender | Participant’s gender | Binary | - 0 = Male - 1 = Female |
| Happy | Happiness following induction | Ordinal | - 1-4 |
| ID | Participant identifier | Nominal | - 1-320 (exp. 1) - 1-203 (exp. 2) |
| IGbfDG | Order indicator of investment game before dictator game | Binary | - 0 = No - 1 = Yes |
| IGbfTDG | Order indicator of investment game before triple dictator game | Binary | - 0 = No - 1 = Yes |
| IGexpect | Amount expected in return in the investment game | Continuous | - 0-30 |
| IGexpectProp | Proportion expected in return in the investment game | Proportion | - 0-6.67 |
| IGgive | Amount given in the investment game | Ordinal | - 0-10 |
| IGrecip# | Amount reciprocated in the investment game for each offer # | Ordinal/  Continuous | - 0-30 |
| IGrecipAvg | Average proportion reciprocated in the investment game for all offers | Proportion | - 0-1 |
| IGrecipProp# | Proportion reciprocated in the investment game for each offer # | Proportion | - 0-1 |
| Memory# | Whether post-induction memory question # was answered correctly | Binary | - 0 = No - 1 = Yes |
| MemoryScore | Total number of correct answers to memory questions | Ordinal | - 1-5 |
| Order | Indicator of order of gameplay | Nominal | - 1 = DG/TDG/IG - 2 = DG/IG/TDG - 3 = - 4 = - 5 = - 6 = |
| Origin | UK or international status | Binary | - 0 = UK - 1 = International |
| Proud | Pride following induction | Ordinal | - 1-4 |
| QCAE# | QCAE questionnaire item # | Ordinal | - 1-4 |
| QualResponse | Qualitative (text) response to why participants’ chose to make their decisions (exp. 2) | Text | - N/A |
| rQCAE# | QCAE questionnaire item # (reversed) | Ordinal | - 1-4 |
| rTrust# | Response to trust question # (reversed) | Ordinal | - 1-4 |
| Sad | Sadness following induction | Ordinal | - 1-4 |
| TDGbfDG | Order indicator of triple dictator game before dictator game | Binary | - 0 = No - 1 = Yes |
| TDGbfIG | Order indicator of triple dictator game before investment game | Binary | - 0 = No - 1 = Yes |
| TDGgive | Amount given in the triple dictator game | Ordinal | - 0-10 |
| Time | Time taken (minutes) | Continuous | - 6-1272 |
| Trust# | Response to trust question # | Ordinal | - 1-4 |
| TrustScore | Total trust for recipient | Continuous | - 3-12 |
| WhichPlayer | Response to please tell us whether you were player 1 / player 2 / unsure (exp. 2) | Nominal | - 1 = Player 1 - 2 = Player 2 - 3 = Unsure |
