# Supplementary material for: The effects of exposure to images of others' suffering and vulnerability on altruistic, trust-based, and reciprocated economic decision-making
Source: PLoS One. 2018 Mar 21;13(3):e0194569. doi: 10.1371/journal.pone.0194569 (PMC5862494; doi:10.1371/journal.pone.0194569)
Supplement: S3 Text — (DOCX) [file pone.0194569.s003.docx]

**Supporting Information (S3)**

**Associations between affective profiles and primary economic outcomes**

**Table S3.1. Levels of the five emotions by condition.**

|  | Compassion | | Sadness | | Disgust | | Happiness | | Pride | |
| --- | --- | --- | --- | --- | --- | --- | --- | --- | --- | --- |
| Level | 0 | 1 | 0 | 1 | 0 | 1 | 0 | 1 | 0 | 1 |
| 1 | 62 | 4 | 148 | 12 | 234 | 62 | 10 | 95 | 106 | 191 |
| 2 | 92 | 32 | 88 | 58 | 17 | 84 | 51 | 133 | 98 | 58 |
| 3 | 93 | 123 | 22 | 137 | 8 | 96 | 182 | 30 | 51 | 11 |
| 4 | 12 | 101 | 1 | 53 | 0 | 18 | 16 | 2 | 4 | 0 |

*N* control (0) = 259. *N* experimental (1) = 260. Level uses a 4-point ordinal scale (1 = *not at all*, 4 = *very*).

**Table S3.2. Pearson correlation matrix between emotions and economic behaviour (pooled).**

|  | (1) | (2) | (3) | (4) | (5) | (6) | (7) | (8) | (9) |
| --- | --- | --- | --- | --- | --- | --- | --- | --- | --- |
| (1) Compassion | - |  |  |  |  |  |  |  |  |
| (2) Sad | .46*** | - |  |  |  |  |  |  |  |
| (3) Disgust | .40*** | .61*** | - |  |  |  |  |  |  |
| (4) Happy | -.28*** | -.69*** | -.51*** | - |  |  |  |  |  |
| (5) Proud | .05 | -.35*** | -.25*** | .45*** | - |  |  |  |  |
| (6) DGgive | .02 | .08† | .09* | -.02 | -.08† | - |  |  |  |
| (7) TDGgive | .09* | .10* | .14** | -.06 | -.07 | .37*** | - |  |  |
| (8) IGgive | .08† | .08† | .06 | -.01 | -.02 | .24*** | .32*** | - |  |
| (9) IGrecipAvg | .05 | .08† | .06 | -.11* | .00 | .22*** | .17*** | .12* | - |

*N* = 519. †*p* < .10. **p* < .05. ***p* < .01. ****p* < .001.

**Table S3.3. Pearson correlation matrix between emotions and economic behaviour (within condition).**

|  | (1) | (2) | (3) | (4) | (5) | (6) | (7) | (8) | (9) |
| --- | --- | --- | --- | --- | --- | --- | --- | --- | --- |
| (1) Compassion | - | -.08 | -.01 | .39*** | .54*** | -.05 | .05 | .08 | -.06 |
| (2) Sad | .38*** | - | .36*** | -.40*** | -.08 | -.12† | .01 | .01 | -.02 |
| (3) Disgust | .15* | .32*** | - | -.21*** | .06 | -.04 | .02 | -.06 | .03 |
| (4) Happy | -.23*** | -.51*** | -.19** | - | .30*** | .13* | .04 | .04 | .04 |
| (5) Proud | -.10 | -.27*** | -.12† | .37*** | - | -.04 | .00 | -.03 | .10 |
| (6) DGgive | .03 | .19** | .12† | -.08 | -.10 | - | .41*** | .31*** | .23*** |
| (7) TDGgive | .00 | .03 | .11† | .01 | -.06 | .32*** | - | .31*** | .17** |
| (8) IGgive | .02 | .07 | .07 | .03 | .06 | .17** | .32*** | - | .20** |
| (9) IGrecipAvg | .08 | .08 | .01 | -.17** | -.06 | .20** | .16** | .04 | - |

*N* control condition (above diagonal) = 259. *N* experimental condition (below diagonal) = 260. †*p* < .10. **p* < .05. ***p* < .01. ****p* < .001.

**Table S3.4. Exploratory multivariate regression output.**

|  | DGgive | | TDGgive | | IGgive | | IGrecipAvg | |
| --- | --- | --- | --- | --- | --- | --- | --- | --- |
|  | 0 | 1 | 0 | 1 | 0 | 1 | 0 | 1 |
| Compassion | -.108 | -.140 | .066 | -.114 | .276† | -.085 | -.255** | .080 |
| Sadness | -.263 | .415* | .016 | .056 | .131 | .270 | -.036 | .001 |
| Disgust | .079 | .067 | .007 | .150 | -.222 | .099 | .069 | -.077 |
| Happiness | .345 | .123 | .261 | .022 | .042 | .120 | .087 | -.211† |
| Pride | -.096 | -.221 | -.183 | -.226 | -.218 | .234 | .273** | .056 |

*N* control condition (0) = 259. *N* experimental condition (1) = 260. Coefficients for DGgive, TDGgive, and IGgive are from ordered logistic regression, and the coefficients for IGrecipAvg are from beta regression. These are log-odds coefficients. †*p* < .10. **p* < .05. ***p* < .01.
