# Supplementary figures and images for: The effects of exposure to images of others' suffering and vulnerability on altruistic, trust-based, and reciprocated economic decision-making
Source: PLoS One. 2018 Mar 21;13(3):e0194569. doi: 10.1371/journal.pone.0194569 (PMC5862494; doi:10.1371/journal.pone.0194569)

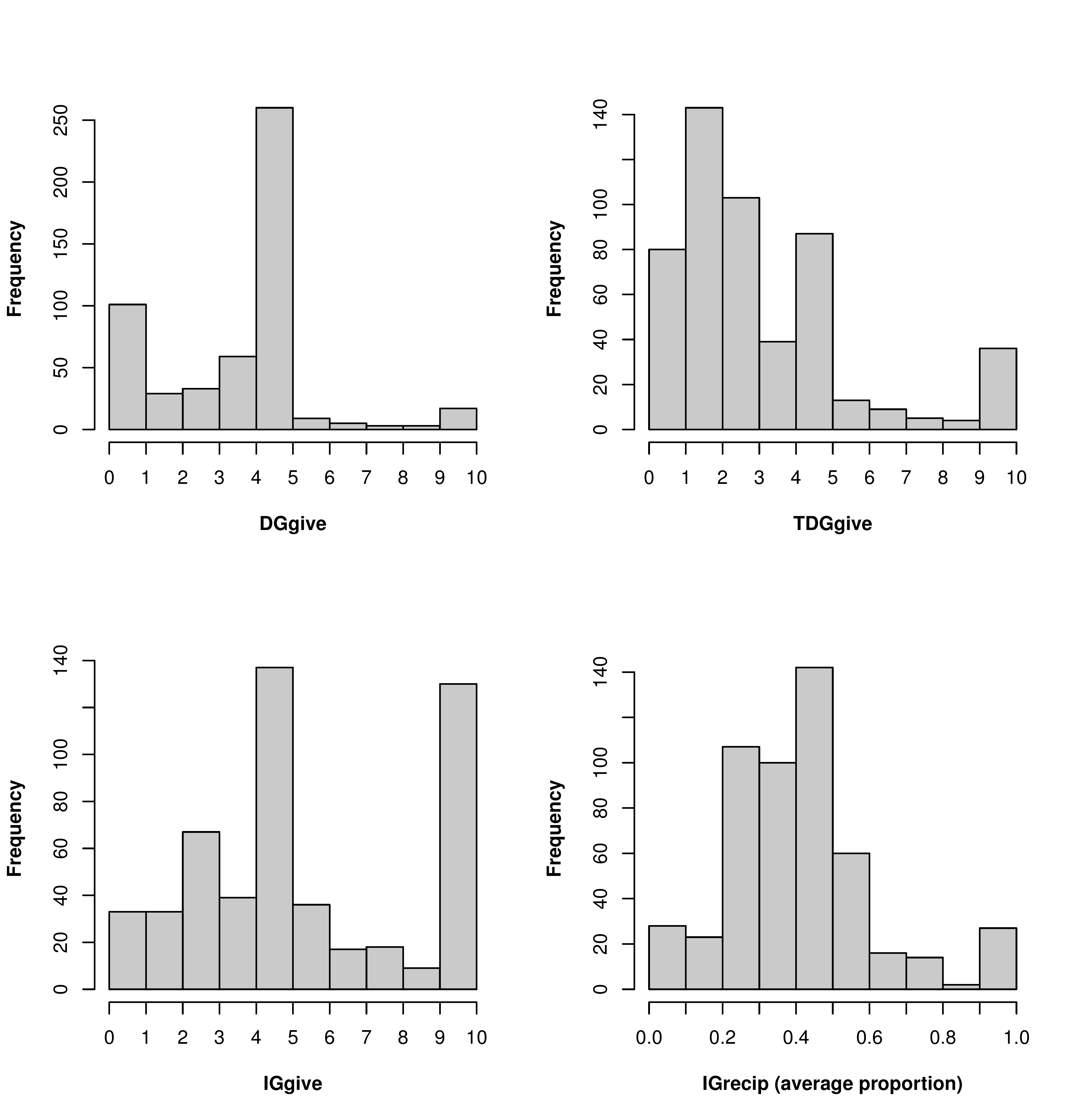

Supplement: S1 Fig — (TIF) [file pone.0194569.s004.tif]
